# Distinct Associations of PTEN and TMPRSS4 Expression with Clinical Outcomes and Fudan Immunohistochemistry-Based Subtypes in Triple-Negative Breast Cancer

**DOI:** 10.3390/life16091563

**Published:** 2026-09-17

**Authors:** Wei Hou, Sheng Zhang, Xianbin Wei, Lixin Zhou, Xinting Diao, Yiqiang Liu, Xiuli Ma

**Affiliations:** 1 Key Laboratory of Carcinogenesis and Translational Research (Ministry of Education), Department of Pathology, Peking University Cancer Hospital & Institute, Beijing 100142, China; houwei112030@163.com (W.H.); jordanzs@126.com (S.Z.); 13601071901@163.com (L.Z.); 15116951640@163.com (X.D.); 2 State Key Laboratory of Holistic Integrative Management of Gastrointestinal Cancers, Peking University Cancer Hospital & Institute, Beijing 100142, China; weixianbin25@stu.pku.edu.cn

**Keywords:** triple-negative breast cancer, PTEN, TMPRSS4, immunohistochemistry, Fudan subtypes, clinical outcomes

## Abstract

Triple-negative breast cancer (TNBC) is a heterogeneous and aggressive subtype with limited therapeutic targets. We retrospectively evaluated the expression of phosphatase and tensin homolog (PTEN) and transmembrane serine protease 4 (TMPRSS4) by immunohistochemistry in formalin-fixed, paraffin-embedded tumor tissues from 145 patients with histologically confirmed TNBC, and we analyzed associations with clinicopathological features, Fudan immunohistochemistry-based subtypes, and clinical outcomes. Clinicopathological analyses included all 145 cases. Exploratory survival analyses included 59 patients with complete, verifiable follow-up and outcome data (median follow-up, 46 months), during which three deaths and eight progression events occurred. PTEN-retained expression was associated with worse overall survival (OS; log-rank *p* = 0.030), whereas TMPRSS4-positive expression was associated with worse progression-free survival (PFS; log-rank *p* = 0.034). TMPRSS4 expression showed a nominal association with Fudan subtype in the unadjusted omnibus analysis (*p* = 0.025), which was considered exploratory after Benjamini–Hochberg correction (q = 0.379). The PTEN and TMPRSS4 staining categories were not significantly associated. In an exploratory four-group survival analysis, patients with concurrent PTEN-retained and TMPRSS4-positive expression (PTEN^+^/TMPRSS4^+^) showed the lowest OS and PFS estimates, patients with PTEN loss and TMPRSS4 negativity (PTEN^−^/TMPRSS4^−^) showed the highest estimates, and the two single-positive groups showed intermediate estimates. Independent transcript-level validation restricted to TNBC in The Cancer Genome Atlas Breast Invasive Carcinoma (TCGA-BRCA) and Molecular Taxonomy of Breast Cancer International Consortium (METABRIC) did not reproduce these protein-level associations. These protein-level findings should therefore be considered exploratory and hypothesis-generating and warrant confirmation in larger, adequately powered cohorts using standardized immunohistochemistry with complete treatment data.

## 1. Introduction

Breast cancer remains the most commonly diagnosed malignancy among women worldwide and constitutes a leading cause of cancer-related mortality. According to Global Cancer Observatory (GLOBOCAN) 2022 estimates, female breast cancer accounts for approximately 2.3 million new cases and 666,000 deaths annually, representing 11.6% of all new cancer cases and 6.9% of cancer-related deaths globally [1,2]. Among the various molecular subtypes, triple-negative breast cancer (TNBC) is defined by the absence of estrogen receptor (ER), progesterone receptor (PR), and human epidermal growth factor receptor 2 (HER2) expression and accounts for 11% to 20% of all breast cancer cases [3]. The Fudan classification, which was established based on comprehensive genomic and transcriptomic profiling of Chinese TNBC cohorts, classifies TNBC into four molecular subtypes—immunomodulatory (IM), luminal androgen receptor (LAR), basal-like immune-suppressed (BLIS), and mesenchymal (MES)—providing a refined framework for subtype-specific therapeutic strategies [4]. Compared with other breast cancer subtypes, TNBC is characterized by a higher histological grade, increased metastatic potential, and limited therapeutic options, resulting in a substantially worse prognosis [5]. Owing to the absence of well-defined molecular targets, conventional chemotherapy has remained a mainstay of treatment for TNBC, with only a proportion of patients achieving substantial clinical benefit [6].

The molecular pathogenesis of TNBC involves complex alterations in multiple signaling pathways. Among these, the phosphoinositide 3-kinase (PI3K)/protein kinase B (AKT)/mechanistic target of rapamycin (mTOR) signaling cascade represents one of the most important regulatory networks involved in the initiation and progression of TNBC [7]. Phosphatase and tensin homolog (PTEN) serves as a pivotal negative regulator of the PI3K/AKT pathway by dephosphorylating phosphatidylinositol (3,4,5)-trisphosphate (PIP3) to phosphatidylinositol (4,5)-bisphosphate (PIP2), thereby attenuating downstream AKT activation [8]. Alterations involving PTEN and the PI3K/AKT pathway are common in TNBC [9]. PTEN loss or mutation leads to constitutive activation of PI3K/AKT signaling, promoting malignant transformation, tumor progression, and therapeutic resistance [10].

Transmembrane serine protease 4 (TMPRSS4), located on chromosome 11q23.3, is a member of the type II transmembrane serine protease family and has emerged as an oncogenic factor in multiple malignancies, including non-small-cell lung cancer, digestive system cancers, prostate cancer, and breast cancer [11,12]. In these malignancies, elevated TMPRSS4 expression has been associated with adverse clinicopathological features, including advanced tumor stage, lymph node metastasis, and poor patient survival [13]. Functionally, TMPRSS4 has been implicated in epithelial–mesenchymal transition, migration, invasion, cancer stem cell-like properties, and modulation of the tumor microenvironment [14].

The relationship between PTEN and TMPRSS4 staining patterns in TNBC has not been well characterized. We therefore evaluated PTEN and TMPRSS4 expression by immunohistochemistry in 145 TNBC cases and examined their associations with clinicopathological features, Fudan immunohistochemistry-based subtypes, and available clinical outcomes. To determine whether these protein-level associations are observed at the transcriptional level, we analyzed PTEN and TMPRSS4 mRNA expression and clinical outcomes in TNBC using TCGA-BRCA and METABRIC data. Together, these analyses suggest that PTEN and TMPRSS4 may provide non-redundant immunophenotypic information and that combined assessment warrants further evaluation as an exploratory prognostic approach in TNBC.

## 2. Materials and Methods

### 2.1. Tissue Selection

The electronic pathology reporting system of Peking University Cancer Hospital was searched for core-needle biopsy specimens reported as triple-negative breast cancer between 2018 and 2024. Pathology sections were reviewed to confirm eligibility. A total of 145 treatment-naïve patients with eligible archived formalin-fixed, paraffin-embedded core-biopsy specimens were identified and included.

Eligible cases were identified according to the following criteria: (1) histologically confirmed primary invasive breast carcinoma; (2) a diagnosis of TNBC based on a pretreatment core-needle biopsy obtained between 2018 and 2024; (3) no systemic treatment before biopsy; (4) availability of an archived formalin-fixed, paraffin-embedded tissue block containing sufficient tumor tissue for immunohistochemical analysis; and (5) availability of the required pathological information. Cases were considered ineligible if they represented recurrent or metastatic disease, if systemic treatment had been administered before biopsy, if the tumor did not meet the diagnostic criteria of TNBC, if receptor-status information was incomplete, or if the available tissue was insufficient for analysis. For patients with more than one biopsy specimen, only one pretreatment specimen was included. The cohort was not strictly consecutive because inclusion required adequate archived tissue; some otherwise eligible cases may therefore have been excluded. Pretreatment core-needle biopsy specimens were selected to assess biomarker expression before therapy and to minimize treatment-related alterations in protein expression.

TNBC was defined as estrogen receptor (ER) and progesterone receptor (PR) expression in <1% of tumor-cell nuclei, together with HER2-negative status. HER2 negativity was defined as an immunohistochemical score of 0 or 1+, or a score of 2+ without HER2 gene amplification by in situ hybridization.

**Figure 1:**
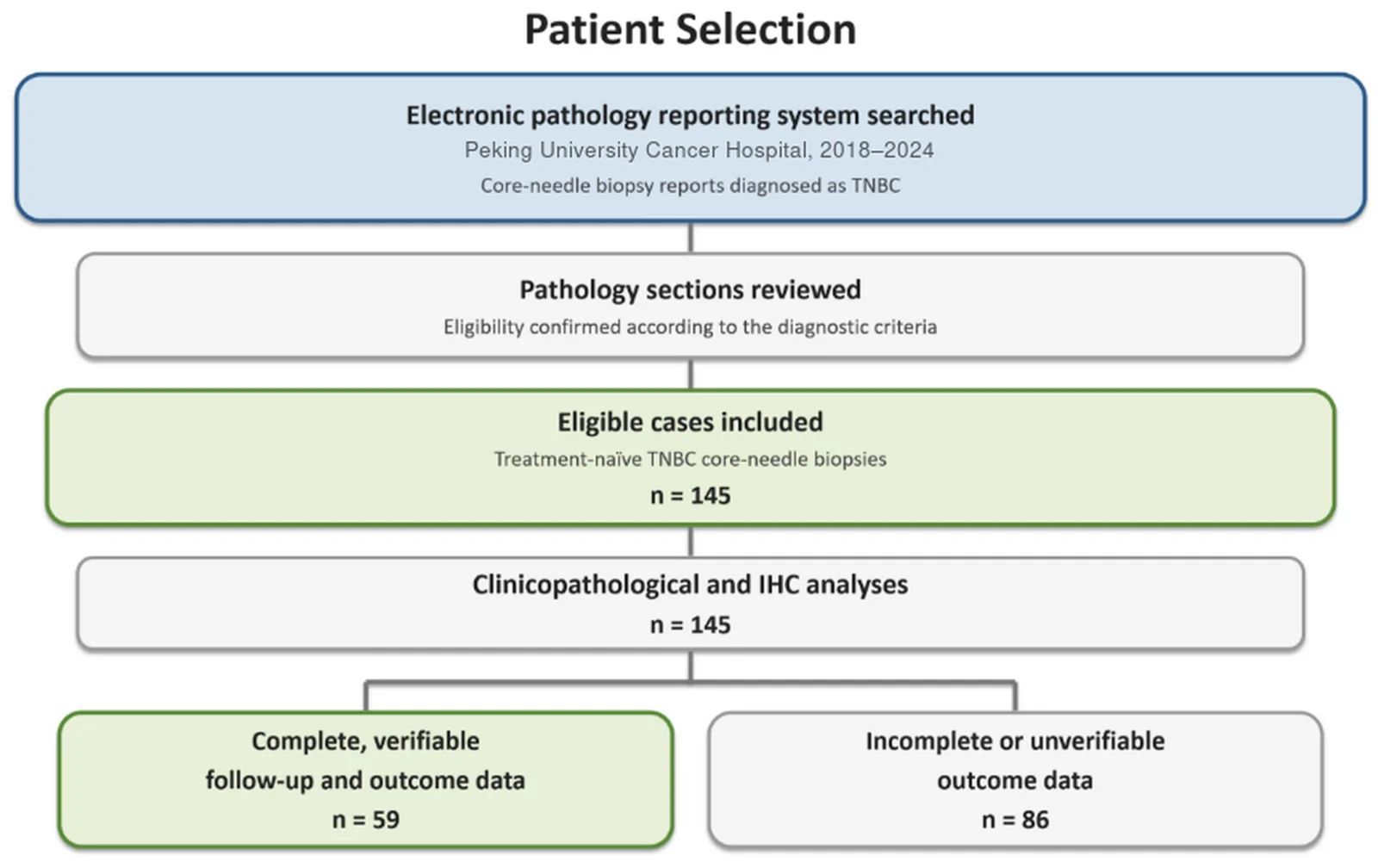
Patient-selection and follow-up-data screening flow diagram for the retrospective TNBC cohort. All 145 eligible cases were included in the clinicopathological and immunohistochemical analyses and underwent follow-up-data screening. Fifty-nine patients had complete, verifiable follow-up and outcome data and were included in the exploratory survival analyses; eight-six patients with incomplete or unverifiable outcome data were excluded only from the survival analyses. IHC, immunohistochemistry; TNBC, triple-negative breast cancer.

The patient-selection and follow-up-data screening processes are summarized in Figure 1.

### 2.2. Immunohistochemical Staining

To establish the optimal working dilutions, 1:100, 1:200, and 1:300 dilutions were tested on TNBC tissue sections; the 1:300 dilution was selected for TMPRSS4. Formalin-fixed, paraffin-embedded blocks were cut into 4 μm sections. The following antibodies were used: PTEN (clone RM265, Gene Technology, Shanghai, China), TMPRSS4 (clone BP6327, Hangzhou Bailing Biotechnology, Hangzhou, China), androgen receptor (AR) (clone EP120, Beijing Zhongshan Jinqiao Biotechnology, Beijing, China), cluster of differentiation 8 (clone SP16, Beijing Zhongshan Jinqiao Biotechnology, Beijing, China), forkhead box C1 (FOXC1) (Gene Technology, Shanghai, China), and doublecortin-like kinase 1 (DCLK1) (Gene Technology, Shanghai, China). All antibodies except anti-TMPRSS4 were used as ready-to-use working solutions, and immunohistochemical staining was performed on a Leica staining platform. Staining followed the manufacturers’ protocols. Antigen retrieval was performed in ethylenediaminetetraacetic acid (EDTA) buffer (pH 9.0) at 95 °C for 36 min.

Known PTEN-retained and TMPRSS4-positive TNBC tissue sections were included as external positive controls for PTEN and TMPRSS4, respectively. Known AR-positive breast carcinoma tissue was used as the external positive control for AR, tonsil tissue as the external positive control for CD8, and previously confirmed FOXC1-positive and DCLK1-positive TNBC tissues as the respective external positive controls. For negative reagent controls, the primary antibody was replaced with antibody diluent while all other staining steps were kept unchanged. Non-neoplastic stromal cells, vascular endothelial cells, and inflammatory cells adjacent to the tumor served as internal positive controls for PTEN, and lymphocytes present in the study sections served as internal positive controls for CD8. Because no fixed internal control was available for TMPRSS4, FOXC1, or DCLK1, staining for these markers was considered technically valid only when the corresponding external positive control showed the expected staining and the negative control remained unstained.

### 2.3. Pathology Evaluation

PTEN expression was categorized as loss or retained. PTEN loss was defined as complete absence of staining in tumor cells in the presence of preserved staining in non-neoplastic internal controls, consistent with previously reported breast cancer PTEN immunohistochemistry scoring approaches [15]. Tumors showing focal, partial, or heterogeneous PTEN loss were not classified as complete loss and were included in the PTEN-retained group. TMPRSS4 expression was categorized as negative or positive. TMPRSS4-positive expression was defined as staining in >10% of tumor cells, irrespective of staining intensity. Tumors with staining in ≤10% of tumor cells were classified as negative. The 10% threshold was used as a study-specific exploratory cutoff to distinguish absent or focal/background-like staining from definite tumor-cell expression and was not treated as a clinically validated cutoff. For the interobserver evaluation, W.H. and Y.L. independently assessed all 145 cases. Each pathologist was blinded to the other reader’s classification and to the clinical outcome information. Agreement statistics were calculated from these two independent pre-consensus readings. For PTEN, the two readers agreed in 129 of 145 cases (89.0%), with Cohen’s κ = 0.779 (95% CI, 0.670–0.875), indicating substantial agreement. For TMPRSS4, the readers agreed in 136 of 145 cases (93.8%), with Cohen’s κ = 0.871 (95% CI, 0.786–0.945), indicating strong agreement. All discordant cases for both markers were jointly re-examined by the two pathologists and resolved by consensus; the consensus classifications were used as the final values for the clinicopathological and survival analyses. Only the categorical reader classifications and final consensus calls were retained, whereas the original staining-percentage and intensity scores were not preserved in a form suitable for reanalysis. Consequently, sensitivity analyses using continuous staining percentages, staining intensity, or H-scores could not be reliably performed.

The Fudan IHC-based classifier was applied hierarchically, as described by Zhao et al. [16]. Cases were assigned in the following order: (1) AR-positive tumors (≥10% tumor-cell staining) were classified as LAR, irrespective of other markers; (2) AR-negative tumors with CD8-positive stromal tumor-infiltrating lymphocytes (≥20%) were classified as IM; (3) AR-negative, CD8-negative tumors with FOXC1 positivity (≥10%) were classified as BLIS; (4) AR-negative, CD8-negative, FOXC1-negative tumors with DCLK1 positivity (≥10%) were classified as MES; and (5) tumors negative for all four markers were classified as unclassified. The assignment hierarchy and thresholds are summarized in Supplementary Table S1. These were the exact thresholds applied to classify all 145 cases in this cohort.

### 2.4. Public Transcript-Level Validation

To assess whether the exploratory protein-level findings were recapitulated at the transcript level, PTEN and TMPRSS4 expression levels were evaluated in TCGA-BRCA (https://portal.gdc.cancer.gov/) and METABRIC (https://www.cbioportal.org/study/summary?id=brca_metabric) (data accessed on 4 September 2026), including TNBC subsets. TNBC was defined as estrogen receptor (ER)-negative, progesterone receptor (PR)-negative, and human epidermal growth factor receptor 2 (HER2)-negative status. Gene expression levels of PTEN and TMPRSS4 were assessed as mRNA abundance: TCGA-BRCA data were quantified as TPM (unstranded) from RNA-seq, and METABRIC data were quantified as microarray signal intensity. For each gene, dataset-specific tertiles were computed to define expression thresholds. In TCGA-BRCA, the 33.3% and 66.7% cutoffs were 17.038 and 27.950 TPM for PTEN, and 0.0871 and 0.7512 TPM for TMPRSS4; in METABRIC, the corresponding cutoffs were 9.393 and 9.835 for PTEN, and 5.466 and 5.622 for TMPRSS4. Patients were accordingly classified into Low, Medium, and High expression groups. Owing to marked platform divergence, tertile thresholds were derived independently within each cohort, and direct numeric comparison of absolute expression values across datasets was avoided.

### 2.5. Statistical Analysis

Categorical variables are presented as *n* (%). For the clinicopathological association analyses, Fisher’s exact test was used for 2 × 2 tables, Pearson’s chi-square test for non-sparse larger tables, and a fixed-margin Monte Carlo exact procedure with 100,000 replicates for larger tables containing expected counts < 5. Effect size was summarized using Cramér’s V with 95% confidence intervals. The baseline comparison between the 59 patients in the survival cohort and the remaining 86 patients (Supplementary Table S2) was descriptive; its *p* values were not included in the multiplicity adjustment. Raw two-sided *p* values are reported, and the Benjamini–Hochberg procedure was applied across the 15 exploratory association tests to control the false-discovery rate; both raw *p* values and adjusted *q* values are provided in Supplementary Table S3. Missing data were not imputed. Clinicopathological and cross-marker association analyses were prespecified; all survival analyses were exploratory.

All 145 patients underwent screening of available follow-up information. Complete, verifiable follow-up and outcome data were available for 59 patients, who were included in the survival cohort irrespective of follow-up duration or event status (a 60-month follow-up was not required); the remaining 86 patients had incomplete or unverifiable outcome information and were excluded from the survival analyses only. Detailed reasons for incomplete or unverifiable outcome information in the remaining 86 patients could not be reliably reconstructed from the retrospective records. Overall survival (OS) was measured from the pathological diagnosis date of the pretreatment core-needle biopsy to death from any cause or censoring. Progression-free survival (PFS) was measured from the same origin to the first documented progression or censoring; deaths without documented progression were not coded as PFS events. Outcome information was obtained from the available follow-up records and telephone follow-up, including progression status, vital status, event dates, and the date of last contact. Patients without an event were censored at their actual recorded last follow-up time. The primary survival analyses within the 59-patient cohort used all available follow-up, without truncation at 60 months. An additional post hoc exploratory 5-year summary administratively censored observations extending beyond 60 months at the 60-month time point while retaining all 59 patients. The last recorded follow-up date was 29 April 2026, and median follow-up estimated by the reverse Kaplan–Meier method was 46 months. For the exploratory four-group survival analysis, the PTEN and TMPRSS4 staining categories were cross-classified to define four mutually exclusive groups: PTEN^−^/TMPRSS4^−^ (PTEN loss with TMPRSS4 negativity), PTEN^−^/TMPRSS4^+^ (PTEN loss with TMPRSS4 positivity), PTEN^+^/TMPRSS4^−^ (PTEN retained with TMPRSS4 negativity), and PTEN^+^/TMPRSS4^+^ (PTEN retained with TMPRSS4 positivity). Kaplan–Meier curves, two-sided log-rank tests, and univariable Cox models were used. Complete treatment data were unavailable, and only three deaths and eight progression events were observed. Consequently, reliable multivariable adjustment for treatment and other clinicopathological covariates was not feasible because such models would produce unstable and overfitted estimates. All survival analyses were therefore treated as exploratory and unadjusted.

All statistical tests were two-sided, and a two-sided *p* value < 0.05 was considered statistically significant. Statistical analyses were performed using IBM SPSS Statistics, version 21.0 (IBM Corp., Armonk, NY, USA).

## 3. Results

### 3.1. Clinicopathological Characteristics of Patients and PTEN and TMPRSS4 Expression

**Figure 2:**
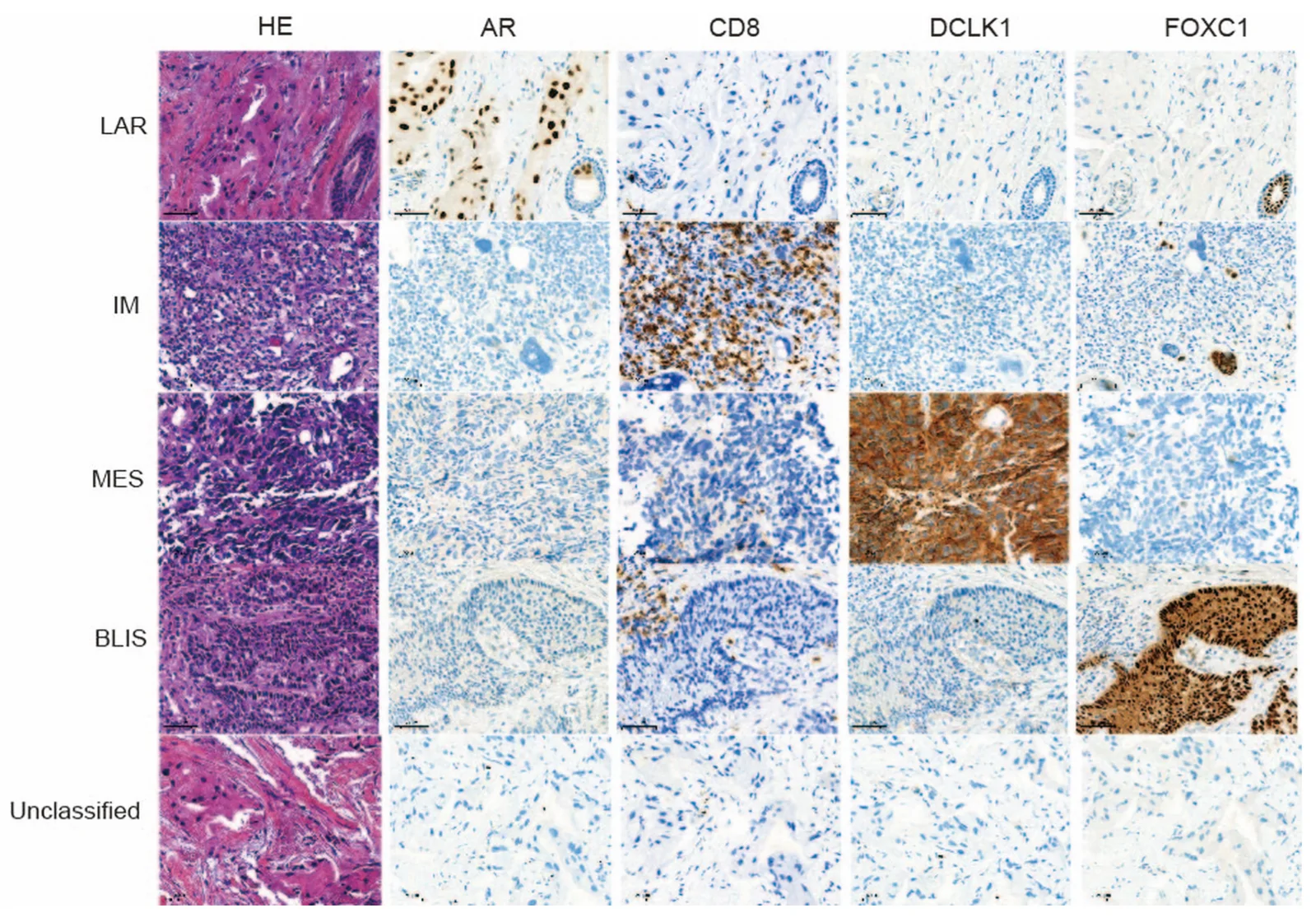
Immunohistochemistry-based Fudan subtypes of triple-negative breast cancer (TNBC). In the immunohistochemistry (IHC) panels, brown chromogenic staining indicates positive marker expression, while nuclei are counterstained blue with hematoxylin. HE, hematoxylin and eosin; AR, androgen receptor; CD8, cluster of differentiation 8; DCLK1, doublecortin-like kinase 1; FOXC1, forkhead box C1. All panels: original magnification, ×40; scale bar, 50 μm.

**Figure 3:**
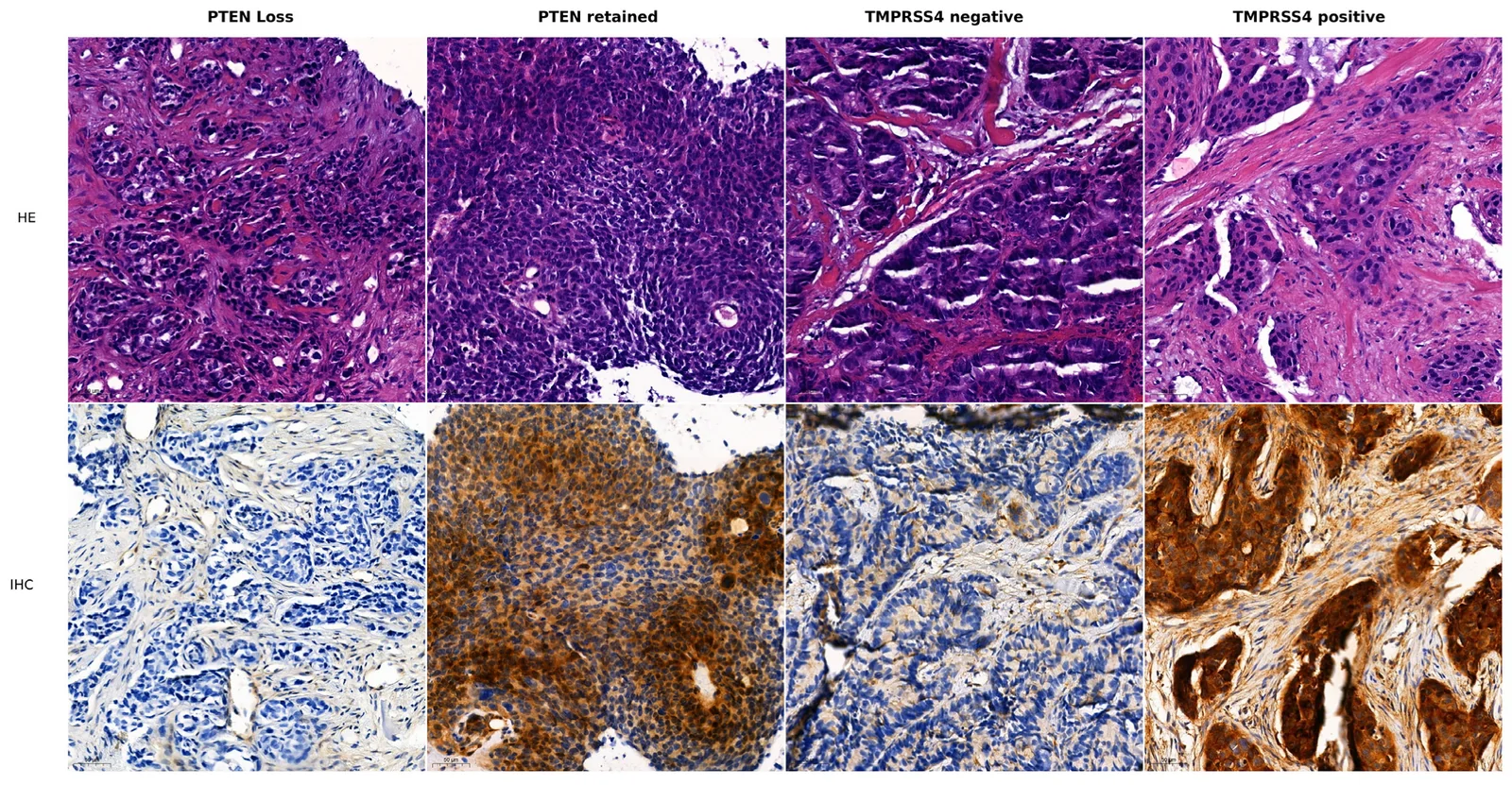
Representative images of PTEN and TMPRSS4 expression in TNBC. In the immunohistochemistry (IHC) panels, brown chromogenic staining indicates positive target expression, while nuclei are counterstained blue with hematoxylin. HE, hematoxylin and eosin; IHC, immunohistochemistry. All panels: original magnification, ×40; scale bar, 50 μm.

A total of 145 patients with TNBC diagnosed at Peking University Cancer Hospital between 2018 and 2024 were included. All specimens were core-needle biopsies obtained before treatment. Age at diagnosis ranged from 24 to 78 years (median, 53 years). At diagnosis, 45 patients (31.0%) were premenopausal and 98 (67.6%) were postmenopausal; menopausal status was unclassified in two cases. Clinical tumor stage was T1 in 43 cases (29.7%), T2 in 96 (66.2%), and T3 in 6 (4.1%). Ninety-three patients (64.1%) had node-negative disease, 51 (35.2%) had node-positive disease, and one was unclassified. Histological types included invasive ductal carcinoma of no special type (138/145, 95.2%), apocrine carcinoma (4/145, 2.8%), adenoid cystic carcinoma (1/145, 0.7%), invasive micropapillary carcinoma (1/145, 0.7%), and mucinous papillary carcinoma (1/145, 0.7%). Histological grade was 2 in 32 cases (22.1%) and 3 in 108 (74.5%); five cases were unclassified. The Fudan IHC-based subtypes were basal-like immune-suppressed (BLIS; 19/145, 13.1%), immunomodulatory (IM; 88/145, 60.7%), luminal androgen receptor (LAR; 34/145, 23.4%), mesenchymal (MES; 2/145, 1.4%), and unclassified (2/145, 1.4%). PTEN expression was lost in 80 tumors (55.2%) and retained in 65 (44.8%). TMPRSS4 expression was negative in 61 tumors (42.1%) and positive in 84 (57.9%) (Table 1; Figure 2 and Figure 3).

### 3.2. Associations of PTEN Expression with Clinicopathological Features and Clinical Outcomes

PTEN status was not significantly associated with age, menopausal status, T stage, nodal status, histological subtype, histological grade, or Fudan IHC-based subtype (all *p* > 0.05, Table 2).

**Figure 4:**
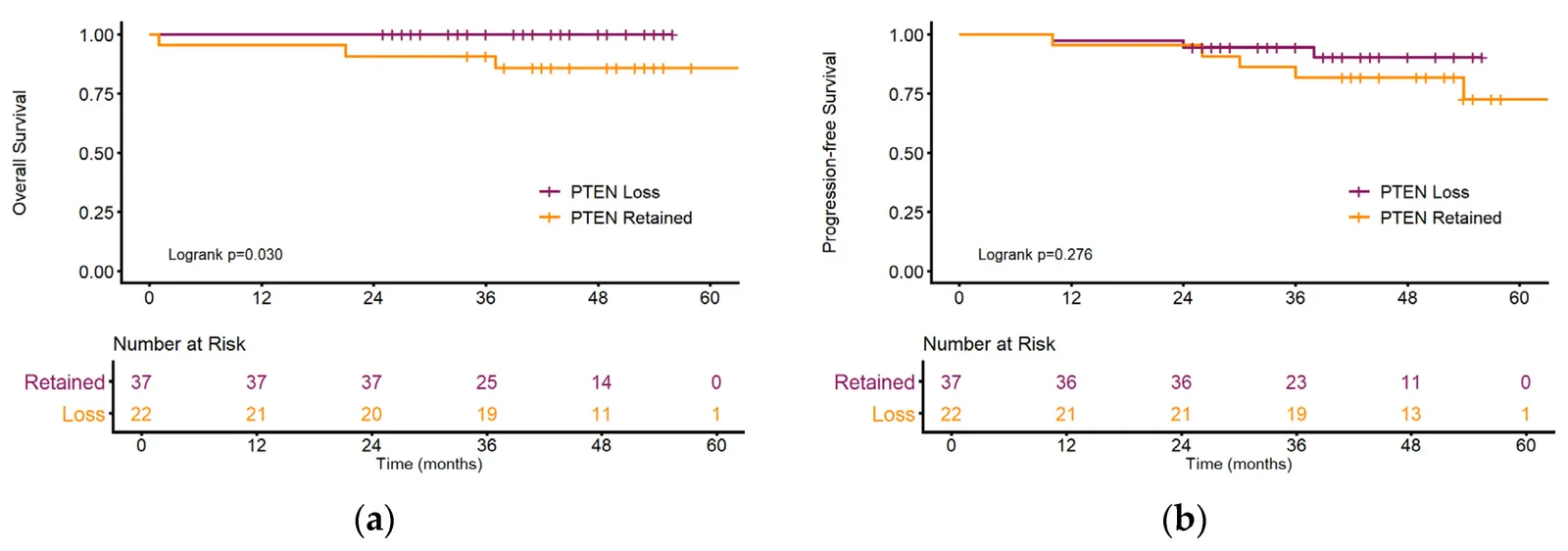
Unadjusted exploratory Kaplan–Meier curves using available follow-up in the 59-patient survival cohort according to PTEN expression status: (**a**) OS (three deaths, all in the PTEN-retained group; log-rank *p* = 0.030; conventional Cox HR not estimable because the PTEN-loss group had no deaths) and (**b**) PFS (eight events; log-rank *p* = 0.276; HR for retained versus loss expression, 2.20; 95% CI, 0.51–9.48). Tick marks indicate censoring and numbers at risk are shown below the plots. OS, overall survival; PFS, progression-free survival; PTEN, phosphatase and tensin homolog. The purple and orange curves represent the PTEN-loss and PTEN-retained groups, respectively.

The exploratory survival cohort included 59 patients with a median follow-up of 46 months; three deaths occurred during follow-up. All three deaths occurred in the PTEN-retained group, so PTEN-retained expression was associated with worse OS (log-rank *p* = 0.030); a conventional Cox HR was not estimable because of complete event separation. PTEN status was not associated with PFS (eight events; log-rank *p* = 0.276; HR for retained versus loss expression, 2.20; 95% CI, 0.51–9.48) (Figure 4 and Supplementary Table S4).

The estimated 5-year overall survival (OS) rate was 94.3% (95% CI, 88.2–100.0%), and the estimated 5-year progression-free survival (PFS) rate was 79.0% (95% CI, 63.8–97.9%). Because few patients remained at risk at 60 months, both estimates should be interpreted with caution.

### 3.3. Associations of TMPRSS4 Expression with Clinicopathological Features and Clinical Outcomes

TMPRSS4 status was not associated with age, menopausal status, T stage, nodal status, histological subtype, or histological grade (all *p* > 0.05). By contrast, TMPRSS4 expression differed across Fudan IHC-based subtypes in a fixed-margin Monte Carlo exact omnibus analysis (*p* = 0.025; Cramér’s V = 0.26; 95% CI, 0.00–0.39), an approach necessitated by the unbalanced subtype distribution (88 IM, 34 LAR, 19 BLIS, 2 MES and 2 unclassified). After Benjamini–Hochberg adjustment, this association did not remain statistically significant (q = 0.379), and the subtype-related pattern is therefore best regarded as an exploratory observation. No subtype-specific post hoc comparisons were performed because of the sparse MES category (*n* = 2), so interpretation was restricted to overall variation in TMPRSS4 expression across Fudan subtypes (Table 3 and Supplementary Table S3).

**Figure 5:**
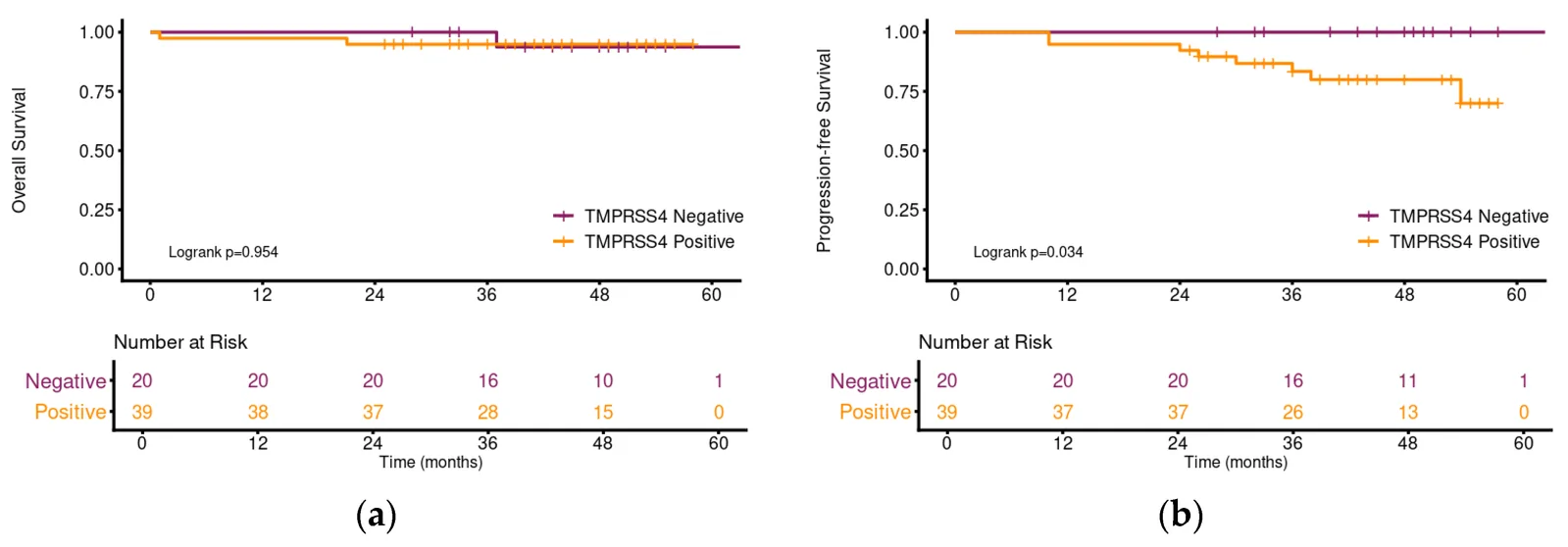
Unadjusted exploratory Kaplan–Meier curves using available follow-up in the 59-patient survival cohort according to TMPRSS4 expression status: (**a**) OS (three deaths; log-rank *p* = 0.954; HR for positive versus negative expression, 1.07; 95% CI, 0.10–11.84) and (**b**) PFS (eight events, all in the TMPRSS4-positive group; log-rank *p* = 0.034; conventional Cox HR not estimable because the negative group had no events). Tick marks indicate censoring and numbers at risk are shown below the plots. OS, overall survival; PFS, progression-free survival; TMPRSS4, transmembrane serine protease 4. The purple and orange curves represent the TMPRSS4-negative and TMPRSS4-positive groups, respectively.

In the same 59-patient survival cohort, TMPRSS4-positive expression was associated with poorer progression-free survival (eight events, all in the positive group; *p* = 0.034). A conventional Cox HR was not estimable because the TMPRSS4-negative group had no PFS events. TMPRSS4 status was not associated with OS (three deaths; log-rank *p* = 0.954; HR for positive versus negative expression, 1.07; 95% CI, 0.10–11.84; *p* = 0.954) (Figure 5 and Supplementary Table S4).

### 3.4. Association Between PTEN and TMPRSS4 Expression and Combined Clinical Outcome Analysis

PTEN and TMPRSS4 staining status were not significantly associated (Fisher’s exact *p* = 0.736; Cramér’s V = 0.04 (95% CI, 0.00–0.20); q = 0.810; Table 4 and Supplementary Table S3). Thus, the two markers may provide distinct rather than redundant information for the immunophenotypic characterization of TNBC.

**Figure 6:**
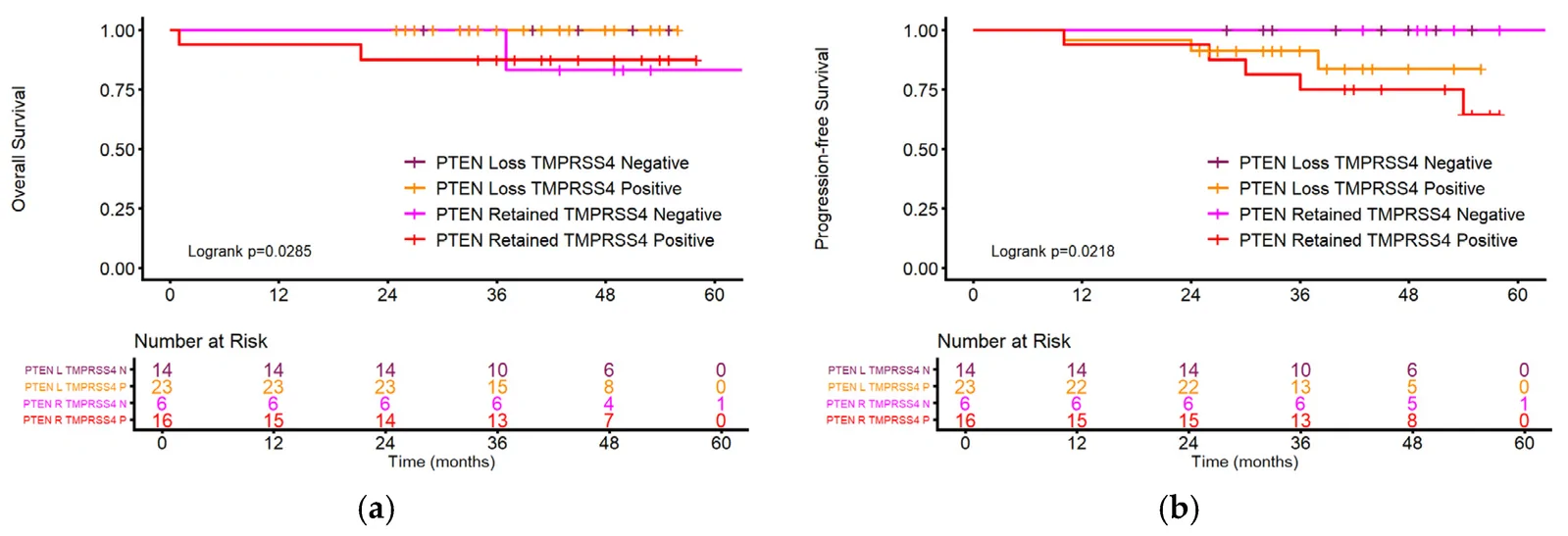
Unadjusted exploratory Kaplan–Meier curves using available follow-up in the 59-patient survival cohort according to PTEN and TMPRSS4 expression status: (**a**) OS (log-rank *p* = 0.029) and (**b**) PFS (log-rank *p* = 0.022). Tick marks indicate censoring and numbers at risk are shown below the plots. OS, overall survival; PFS, progression-free survival; TMPRSS4, transmembrane serine protease 4. The colored curves correspond to the four PTEN/TMPRSS4 expression groups indicated in the figure legend.

Building on the single-marker analyses above, we next examined whether this non-redundant information is reflected in clinical outcomes and whether combined PTEN/TMPRSS4 status may capture broader outcome-related heterogeneity. In the exploratory four-group survival analyses, patients with concurrent PTEN-retained and TMPRSS4-positive expression (PTEN^+^/TMPRSS4^+^) showed the lowest overall survival (OS) and progression-free survival (PFS) estimates, whereas patients with PTEN loss and TMPRSS4 negativity (PTEN^−^/TMPRSS4^−^) showed the highest estimates, and the two single-positive groups showed intermediate estimates (unadjusted log-rank *p* = 0.029 for OS and 0.022 for PFS; Figure 6a,b). In view of the exploratory design and the number of events available in the survival cohort (three deaths and eight progression events), these analyses should be interpreted as descriptive and hypothesis-generating. Accordingly, the observed survival patterns provide a rationale for evaluating the combined PTEN/TMPRSS4 assessment in larger, adequately powered, treatment-annotated cohorts.

### 3.5. Transcript-Level Validation of PTEN and TMPRSS4 Prognostic Value in Public TNBC Cohorts

**Figure 7:**
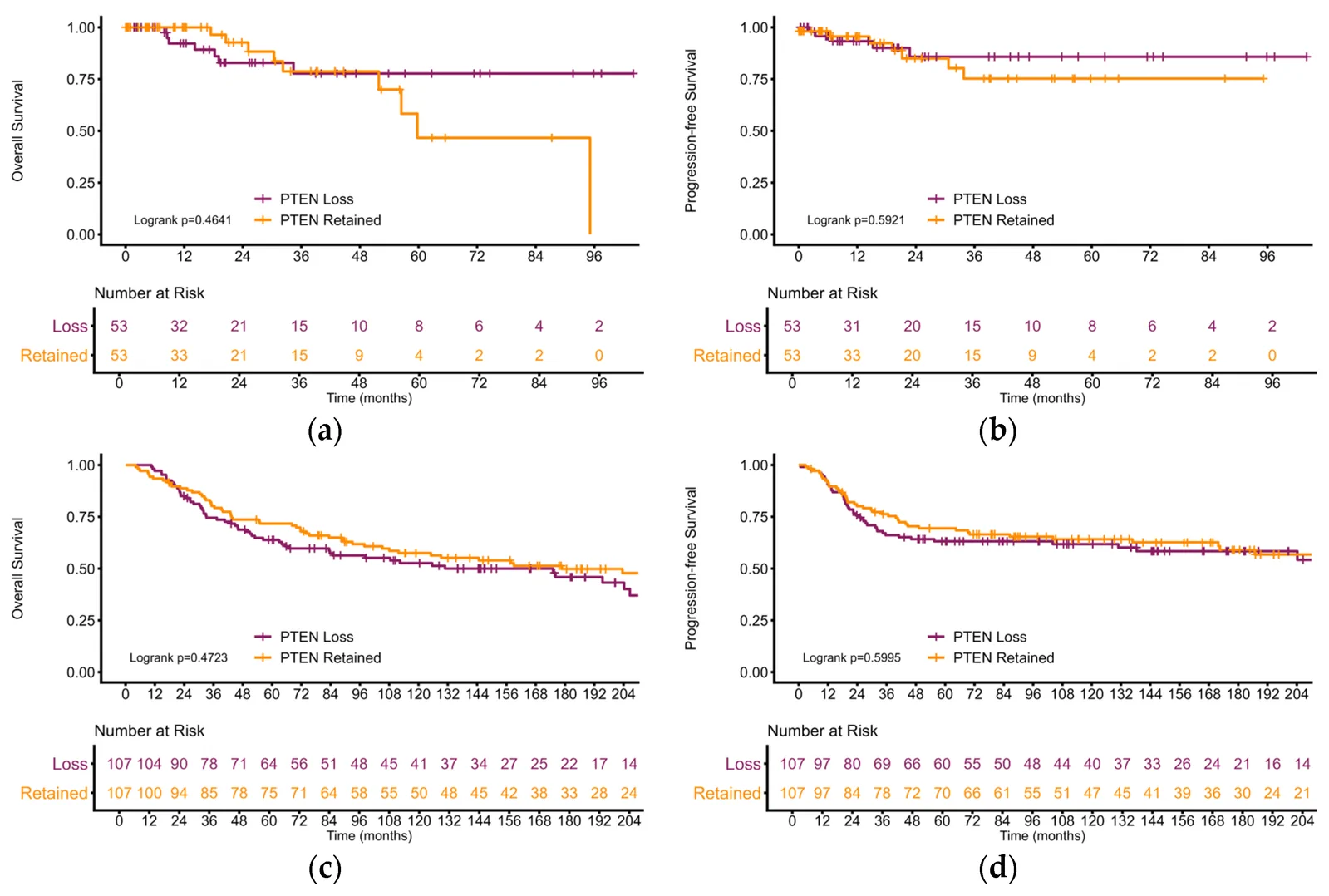
Kaplan–Meier survival analysis of PTEN transcript levels in TNBC. (**a**,**b**) OS (**a**) and PFS (**b**) in the TCGA-BRCA TNBC cohort, comparing the lowest (PTEN Loss, ≤17.038 TPM, *n* = 53) versus highest (PTEN Retained, ≥27.950 TPM, *n* = 53) expression tertiles. (**c**,**d**) OS (**c**) and PFS (**d**) in the METABRIC TNBC cohort, comparing the lowest (PTEN Loss, ≤9.393, *n* = 107) versus highest (PTEN Retained, ≥9.835, *n* = 107) tertiles of log2-transformed expression. *p* values were calculated by the log-rank test; tick marks indicate censored observations, and numbers at risk are shown below each plot. Analyses are unadjusted and exploratory. The purple and orange curves represent the lower and higher PTEN expression groups, respectively.

**Figure 8:**
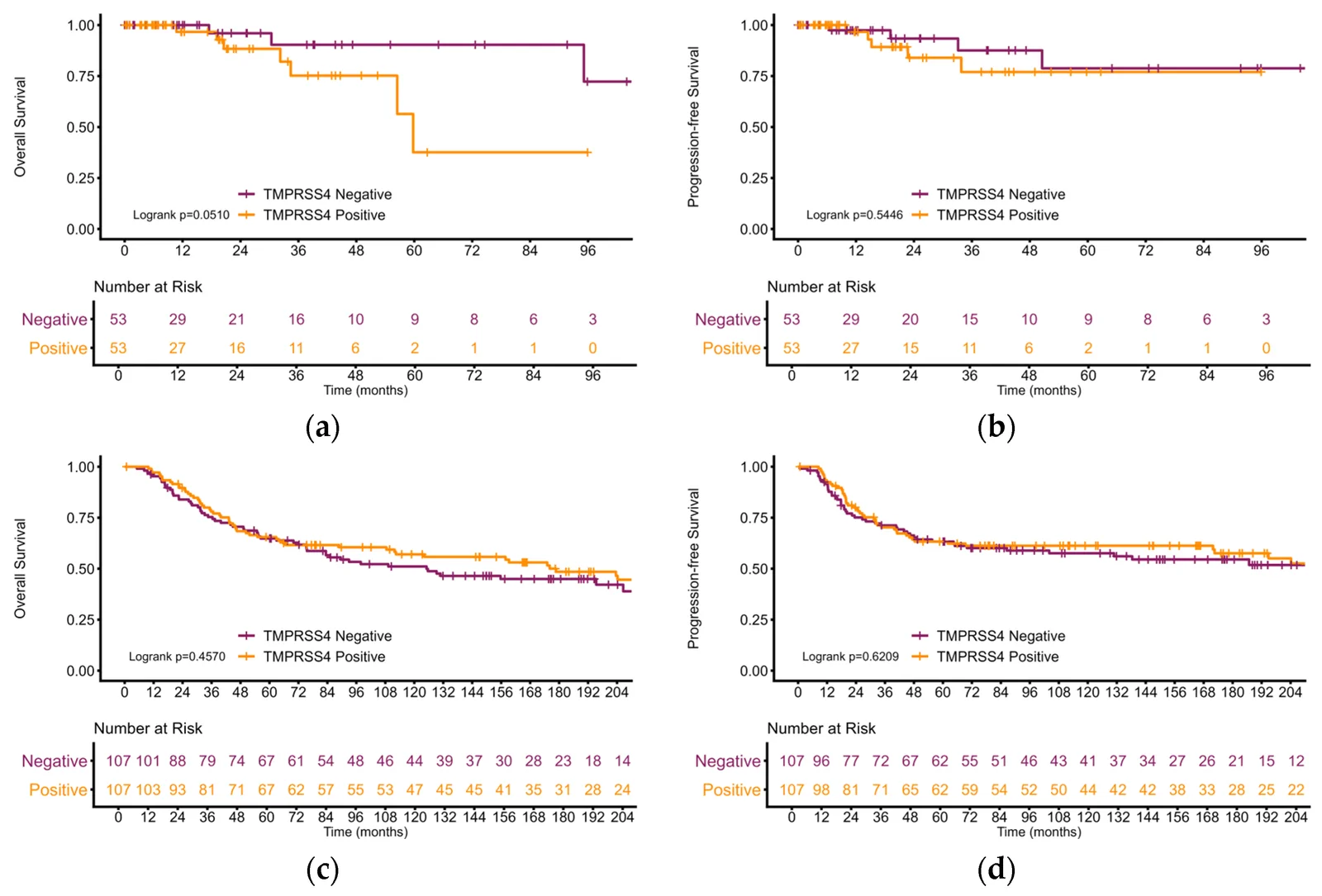
Kaplan–Meier survival analysis of TMPRSS4 transcript levels in TNBC. (**a**,**b**) OS (**a**) and PFS (**b**) in the TCGA-BRCA TNBC cohort, comparing the lowest (TMPRSS4 Negative, ≤0.0871 TPM, *n* = 53) versus highest (TMPRSS4 Positive, ≥0.7512 TPM, *n* = 53) expression tertiles. (**c**,**d**) OS (**c**) and PFS (**d**) in the METABRIC TNBC cohort, comparing the lowest (TMPRSS4 Negative, ≤5.466, *n* = 107) versus highest (TMPRSS4 Positive, ≥5.622, *n* = 107) tertiles of log2-transformed expression. *p* values were calculated by the log-rank test; tick marks indicate censored observations, and numbers at risk are shown below each plot. Analyses are unadjusted and exploratory. The purple and orange curves represent the lower and higher TMPRSS4 expression groups, respectively.

To assess the transcript-level prognostic relevance of PTEN and TMPRSS4, we compared the lowest and highest expression tertiles in two independent TNBC cohorts (TCGA-BRCA, *n* = 106; METABRIC, *n* = 214). PTEN transcript levels were not significantly associated with overall survival (OS) or progression-free survival (PFS) in TCGA-BRCA (*p* = 0.464 and *p* = 0.592) or METABRIC (*p* = 0.472 and *p* = 0.599; Figure 7a–d). In METABRIC, tumors in the lowest PTEN tertile showed numerically higher survival within the first year, but the curves converged thereafter. TMPRSS4 transcript levels were not significantly associated with OS or PFS in either cohort; a nonsignificant trend toward worse OS in TCGA-BRCA emerged after approximately 12 months but was not observed in METABRIC (Figure 8a–d). Overall, transcript-level PTEN and TMPRSS4 expression levels were not significantly associated with survival in these unadjusted exploratory analyses. The transient METABRIC pattern for PTEN and the unreplicated TCGA-BRCA trend for TMPRSS4 therefore warrant confirmation in larger cohorts with multivariable adjustment (Figure 7 and Figure 8; Supplementary Table S5).

## 4. Discussion

Triple-negative breast cancer (TNBC) remains the most aggressive and therapeutically challenging subtype of breast cancer, characterized by extensive inter- and intra-tumoral heterogeneity. Although the Fudan classification captures biologically distinct TNBC subgroups, accessible immunohistochemical markers are still needed to refine prognostic stratification within these subgroups. In this retrospective cohort of 145 treatment-naïve TNBC cases, PTEN loss and TMPRSS4-positive expression were frequent and were not significantly associated with each other. In exploratory analyses, PTEN-retained expression was associated with worse OS, whereas TMPRSS4-positive expression was associated with worse PFS, while TMPRSS4 expression showed only a nominal association with Fudan subtype that did not remain statistically significant after multiple-testing correction. In the exploratory four-group survival analysis, patients with concurrent PTEN-retained and TMPRSS4-positive expression (PTEN^+^/TMPRSS4^+^) showed the lowest overall survival (OS) and progression-free survival (PFS) estimates, whereas those with PTEN loss and TMPRSS4 negativity (PTEN^−^/TMPRSS4^−^) showed the highest estimates. To assess whether these protein-level patterns extended to the transcript level, we evaluated PTEN and TMPRSS4 mRNA expression in TCGA-BRCA and METABRIC TNBC cohorts; neither marker was significantly associated with survival. These transcript-level results therefore position the primary IHC observations as protein-level and exploratory and support the validation of the combined PTEN/TMPRSS4 assessment in larger, adequately powered cohorts with standardized immunohistochemistry and complete treatment data.

PTEN is a critical tumor suppressor that negatively regulates PI3K/AKT/mTOR signaling [17,18]. Although PTEN alterations are common in TNBC [19], their prognostic value remains controversial. Complete PTEN loss has been associated with aggressive features and worse survival in TNBC [20,21], and meta-analytic evidence links PTEN loss with larger tumor size, nodal metastasis, higher grade, and poorer survival in breast cancer [22]; conversely, in the Nurses’ Health Studies, PTEN loss was associated with lower breast cancer-specific mortality in ER-negative tumors (HR for PTEN loss vs. retained, 0.68; 95% CI, 0.48–0.95), suggesting that retained PTEN may identify a higher-risk subset [23]. In our cohort, PTEN loss was more frequent (80/145, 55.2%), but all three deaths occurred in the PTEN-retained group, which was associated with worse OS by unadjusted log-rank testing (*p* = 0.030; Cox HR not estimable). This discrepancy may reflect the imperfect correlation between immunohistochemical PTEN staining and functional PTEN activity, because detectable PTEN can coexist with partial loss, inactivating mutations, post-translational modifications, or downstream PI3K/AKT activation, whereas complete PTEN loss may identify a more homogeneous, PI3K-dependent phenotype with distinct treatment sensitivity. Therefore, PTEN status in TNBC should be interpreted cautiously and in a treatment- and subtype-dependent context; PTEN alone appears insufficient for prognostic stratification, and its combination with TMPRSS4, molecular subtype, and complete treatment data should be evaluated in larger cohorts.

TMPRSS4 is a type II transmembrane serine protease implicated in epithelial–mesenchymal transition, extracellular-matrix remodeling, and invasive behavior in several cancers [11,24]. In breast cancer, TMPRSS4 expression has been associated with aggressive clinicopathological features and poor survival [24]. In the present 59-patient survival cohort, all eight progression events occurred in the TMPRSS4-positive group, and TMPRSS4-positive expression was associated with poorer PFS by unadjusted log-rank testing (*p* = 0.034). A conventional Cox HR could not be estimated because the TMPRSS4-negative group had no events. TMPRSS4 expression showed a nominal association with Fudan immunohistochemistry-based subtype in the unadjusted omnibus analysis (raw *p* = 0.025; Cramér’s V = 0.26; 95% CI, 0.00–0.39), but this association did not remain statistically significant after Benjamini–Hochberg correction (q = 0.379). Because the subtype distribution was highly unbalanced and only two MES cases were available, no subtype-specific post hoc comparisons were performed. Therefore, the observed subtype pattern should be considered exploratory and warrants further investigation in larger cohorts with more balanced subtype representation.

PTEN and TMPRSS4 staining categories were not statistically associated (Fisher’s exact *p* = 0.736), consistent with distinct, non-redundant immunophenotypic information. In the exploratory four-group survival analysis, PTEN^+^/TMPRSS4^+^ tumors showed the lowest overall survival (OS) and progression-free survival (PFS) estimates, PTEN^−^/TMPRSS4^−^ tumors showed the highest estimates, and the two single-positive groups showed intermediate estimates. Together, these patterns suggest that combined PTEN/TMPRSS4 status may carry prognostic information and provide a testable hypothesis for future studies. On the basis of published evidence, PTEN-related PI3K/AKT signaling and TMPRSS4-mediated invasion and epithelial–mesenchymal transition may each contribute to TNBC progression, although whether these processes converge or act independently remains to be established. RACK1 may provide this molecular link, with evidence associating it with breast cancer progression and PI3K/AKT signaling [25,26], AKT regulation in PTEN-deficient prostate models [27], and GR-dependent RACK1 expression and migration in TNBC [28]. This literature-based framework, together with the observed patterns, supports future evaluation of PTEN, TMPRSS4, and RACK1, while emphasizing that the putative biological signal should ultimately be confirmed at the protein level.

In parallel, transcript-level analyses in TNBC from TCGA-BRCA and METABRIC did not reproduce the protein-level associations observed in our cohort. Rather than contradicting the IHC findings, this discrepancy clarifies their interpretative boundaries. Protein abundance is not a direct surrogate for mRNA abundance: PTEN activity can be altered through post-translational modifications, protein stability, subcellular localization, or upstream pathway feedback even when mRNA levels remain unchanged, and transcript-based tertile cutoffs do not correspond to the binary IHC categories used here. Population, subtype annotation, treatment, and follow-up differences between the original and public cohorts may also contribute to divergent results. These considerations reinforce the rationale for validating the combined PTEN/TMPRSS4 assessment using standardized immunohistochemistry in larger, adequately powered cohorts with complete treatment data.

Several limitations should be acknowledged. This retrospective, single-center study restricted survival analyses to 59 of 145 patients with complete, verifiable follow-up and outcome data; although baseline characteristics were broadly comparable between included and nonincluded patients (Supplementary Table S2), selection bias cannot be fully excluded. Only three deaths and eight progression events were available, and complete event separation precluded stable conventional HR estimation. The small event counts and incomplete treatment data also limited multivariable adjustment, so the survival findings should be interpreted as exploratory and hypothesis-generating rather than definitive. The original TMPRSS4 staining-percentage and intensity scores were unavailable, precluding H-score sensitivity analyses; however, categorical TMPRSS4 classification was based on blinded dual-reader consensus with substantial agreement for PTEN and strong agreement for TMPRSS4. Sparse Fudan subtypes, the absence of genomic/functional data, and the retrospective design also limit mechanistic interpretation. Despite these limitations, the independent blinded dual-reader assessment of PTEN and TMPRSS4 in this well-characterized TNBC cohort identifies candidate associations with survival and Fudan subtypes. These findings support the continued evaluation of PTEN and TMPRSS4 as accessible biomarkers and may lead to them being prioritized for future prospective validation.

## 5. Conclusions

In this treatment-naïve TNBC cohort, PTEN loss and TMPRSS4-positive expression were frequent and not statistically associated, suggesting that the two markers may provide non-redundant immunophenotypic information. In exploratory protein-level survival analyses, PTEN-retained expression was associated with worse overall survival, TMPRSS4-positive expression was associated with worse progression-free survival, and the four-group analysis revealed an ordered pattern in which patients with concurrent PTEN-retained and TMPRSS4-positive expression (PTEN^+^/TMPRSS4^+^) showed the lowest overall survival and progression-free survival estimates, whereas those with PTEN loss and TMPRSS4 negativity (PTEN^−^/TMPRSS4^−^) showed the highest estimates. TMPRSS4 expression also varied across Fudan immunohistochemistry-based subtypes, although this association did not remain significant after multiple-testing correction. Independent transcript-level analyses in TCGA-BRCA and METABRIC did not reproduce these protein-level findings, indicating that the observed relationships are protein-level, assay-specific, and hypothesis-generating. Combined PTEN/TMPRSS4 immunohistochemistry nevertheless provides a testable framework for outcome-oriented TNBC research, and validation in larger, adequately powered cohorts with standardized staining and complete treatment data will clarify its potential to complement existing subtype-based approaches.

## Figures and Tables

**Table 1:** Clinical and pathological characteristics of patients.

Variable	Category	*n* (%)
Age, years	<40	22 (15.2%)
	40–59	85 (58.6%)
	≥60	38 (26.2%)
Menopausal status	Premenopausal	45 (31.0%)
	Postmenopausal	98 (67.6%)
	Unclassified	2 (1.4%)
T stage	T1	43 (29.7%)
	T2	96 (66.2%)
	T3	6 (4.1%)
Nodal status	Node-negative	93 (64.1%)
	Node-positive	51 (35.2%)
	Unclassified	1 (0.7%)
Histological subtype	IDC-NST	138 (95.2%)
	AC	4 (2.8%)
	ACC	1 (0.7%)
	IMPC	1 (0.7%)
	MPC	1 (0.7%)
Histological grade	2	32 (22.1%)
	3	108 (74.5%)
	Unclassified	5 (3.4%)
Fudan subtype	BLIS	19 (13.1%)
	IM	88 (60.7%)
	LAR	34 (23.4%)
	MES	2 (1.4%)
	Unclassified	2 (1.4%)
PTEN status	Loss	80 (55.2%)
	Retained	65 (44.8%)
TMPRSS4 status	Negative	61 (42.1%)
	Positive	84 (57.9%)

Abbreviations: IDC-NST, invasive ductal carcinoma of no special type; AC, apocrine carcinoma; ACC, adenoid cystic carcinoma; IMPC, invasive micropapillary carcinoma; MPC, mucinous papillary carcinoma; BLIS, basal-like immune-suppressed; IM, immunomodulatory; LAR, luminal androgen receptor; MES, mesenchymal.

**Table 2:** Association between PTEN expression status and clinicopathological characteristics in patients with triple-negative breast cancer.

Variable	Category	PTEN Loss, *n* (%)	PTEN Retained, *n* (%)	*p* Value
Age, years	≥60	22 (27.5%)	16 (24.6%)	0.810
	<40	13 (16.2%)	9 (13.8%)	
	40–59	45 (56.2%)	40 (61.5%)	
Menopausal status	Premenopausal	30 (38.0%)	15 (23.4%)	0.072
	Postmenopausal	49 (62.0%)	49 (76.6%)	
T stage	T1	21 (26.2%)	22 (33.8%)	0.596
	T2	55 (68.8%)	41 (63.1%)	
	T3	4 (5.0%)	2 (3.1%)	
Nodal status	Node-negative	53 (66.2%)	40 (62.5%)	0.726
	Node-positive	27 (33.8%)	24 (37.5%)	
Histological subtype	AC	2 (2.5%)	2 (3.1%)	0.738
	IDC-NST	77 (96.2%)	61 (93.8%)	
	MPC	1 (1.2%)	0 (0.0%)	
	ACC	0 (0.0%)	1 (1.5%)	
	IMPC	0 (0.0%)	1 (1.5%)	
Histological grade	2	20 (25.6%)	12 (19.4%)	0.423
	3	58 (74.4%)	50 (80.6%)	
Fudan subtype	BLIS	13 (16.2%)	6 (9.2%)	0.781
	IM	45 (56.2%)	43 (66.2%)	
	LAR	20 (25.0%)	14 (21.5%)	
	MES	1 (1.2%)	1 (1.5%)	
	Unclassified	1 (1.2%)	1 (1.5%)	

Note: Data are presented as *n* (%). Some variables have missing values, so subtotals may not equal 100%. *p* values were obtained using Fisher’s exact, Pearson’s chi-square, or fixed-margin Monte Carlo exact tests as appropriate; test-specific effect sizes and Benjamini–Hochberg-adjusted *q* values are reported in Supplementary Table S3.

**Table 3:** Association between TMPRSS4 expression status and clinicopathological characteristics in patients with triple-negative breast cancer.

Variable	Category	TMPRSS4 Negative, *n* (%)	TMPRSS4 Positive, *n* (%)	*p* Value
Age, years	≥60	15 (24.6%)	23 (27.4%)	0.726
	<40	8 (13.1%)	14 (16.7%)	
	40–59	38 (62.3%)	47 (56.0%)	
Menopausal status	Premenopausal	16 (26.2%)	29 (35.4%)	0.278
	Postmenopausal	45 (73.8%)	53 (64.6%)	
T stage	T1	21 (34.4%)	22 (26.2%)	0.282
	T2	39 (63.9%)	57 (67.9%)	
	T3	1 (1.6%)	5 (6.0%)	
Nodal status	Node-negative	44 (73.3%)	49 (58.3%)	0.078
	Node-positive	16 (26.7%)	35 (41.7%)	
Histological subtype	AC	1 (1.6%)	3 (3.6%)	0.714
	ACC	1 (1.6%)	0 (0.0%)	
	IDC-NST	59 (96.7%)	79 (94.0%)	
	IMPC	0 (0.0%)	1 (1.2%)	
	MPC	0 (0.0%)	1 (1.2%)	
Histological grade	2	10 (17.2%)	22 (26.8%)	0.223
	3	48 (82.8%)	60 (73.2%)	
Fudan subtype	BLIS	13 (21.3%)	6 (7.1%)	0.025
	IM	31 (50.8%)	57 (67.9%)	
	LAR	14 (23.0%)	20 (23.8%)	
	MES	1 (1.7%)	1 (1.2%)	
	Unclassified	2 (3.3%)	0 (0.0%)	

Note: Data are presented as *n* (%). Some variables have missing values, so subtotals may not equal 100%. *p* values were obtained using Fisher’s exact, Pearson’s chi-square, or fixed-margin Monte Carlo exact tests as appropriate; test-specific effect sizes and Benjamini–Hochberg-adjusted *q* values are reported in Supplementary Table S3.

**Table 4:** Association between PTEN and TMPRSS4 expression in patients with triple-negative breast cancer.

TMPRSS4 Status	PTEN Loss, *n* (%)	PTEN Retained, *n* (%)	*p* Value
Negative	35 (43.8%)	26 (40.0%)	0.736
Positive	45 (56.2%)	39 (60.0%)	

Note: Fisher’s exact test was used; effect size and adjusted *q* value are reported in Supplementary Table S3.

## Data Availability

The data supporting the findings of this study are available from the corresponding author upon reasonable request. The data are not publicly available due to patient privacy and ethical restrictions.

## Supplementary Materials

The following supporting information can be downloaded at: https://www.mdpi.com/article/10.3390/life16091563/s1, Table S1: Assignment hierarchy and thresholds of the Fudan immunohistochemistry-based classifier; Table S2: Baseline comparison between the 59 patients included in the survival cohort and the remaining 86 patients; Table S3: Exact tests, effect sizes, 95% confidence intervals, and multiplicity-adjusted results for the 15 exploratory categorical association tests; Table S4: Unadjusted survival analyses in the 59-patient cohort; Table S5: Unadjusted survival analyses in the TCGA-BRCA and METABRIC cohorts.
