# Supplementary material for: Distinct Associations of PTEN and TMPRSS4 Expression with Clinical Outcomes and Fudan Immunohistochemistry-Based Subtypes in Triple-Negative Breast Cancer
Source: Life (Basel). 2026 Sep 17;16(9):1563. doi: 10.3390/life16091563 (PMC13608152; doi:10.3390/life16091563)
Supplement: Supplementary file 1 [file life-16-01563-s001.zip › life-4474484-Supplementary.pdf]

Supplementary Table S1. Assignment hierarchy and thresholds of the Fudan immunohistochemistry-based classifier.

Priority	AR	CD8	FOXC1	DCLK1	Subtype
1	≥10%	Any	Any	Any	LAR
2	<10%	≥20%	Any	Any	IM
3	<10%	<20%	≥10%	Any	BLIS
4	<10%	<20%	<10%	≥10%	MES
5	<10%	<20%	<10%	<10%	Unclassified

Table S1 note: Cases were classified in hierarchical order from priority 1 to 5, and each case was assigned the subtype corresponding to the first criterion met. Thresholds for AR, FOXC1, and DCLK1 refer to the percentage of positively stained tumor cells; the CD8 threshold refers to the percentage of stromal tumor-infiltrating lymphocytes. "Any" indicates that the marker did not influence classification at that step. AR-positive tumors were classified as LAR regardless of the other markers; tumors negative for all four markers were classified as unclassified. AR, androgen receptor; LAR, luminal androgen receptor; IM, immunomodulatory; BLIS, basal-like immune-suppressed; MES, mesenchymal; TNBC, triple-negative breast cancer.

Supplementary Table S2. Baseline comparison between the 59 patients included in the survival cohort and the remaining 86 patients.

Variable	Category	Survival cohort (n = 59)	Not included in survival analysis (n = 86)	P value	Effect size (95% CI)
Age	<40	9	13	0.598	V=0.08 (0.00–0.22)
	40–59	32	53		
	≥60	18	20		
T stage	T1	18	25	1.000	V=0.03 (0.00–0.13)
	T2	39	57		
	T3	2	4		
Nodal status	Node-negative	40	53	0.596	V=0.06 (0.00–0.22)
	Node-positive	19	32		
Histological grade	2	15	17	0.410	V=0.08 (0.00–0.25)
	3	40	68		
Histological subtype	AC	1	3	0.453	V=0.17 (0.00–0.28)
	ACC	1	0		
	IDC-NST	56	82		
	IMPC	1	0		
	MPC	0	1		
Fudan subtype	BLIS	6	13	0.349	V=0.18 (0.00–0.30)
	IM	36	52		
	LAR	17	17		
	MES	0	2		
	Unclassified	0	2		

PTEN status	Loss	37	43	0.174	V=0.13 (0.00–0.29)
	Retained	22	43		
TMPRSS4 status	Negative	20	41	0.124	V=0.14 (0.00–0.30)
	Positive	39	45		

Table S2 note: Pearson's chi-square test was used for age; Fisher's exact test for 2 × 2 tables; and fixed-margin Monte Carlo exact testing with 100,000 replicates for sparse larger tables. V, Cramér's V. Missing values were excluded by variable.

Supplementary Table S3. Exact tests, effect sizes, 95% confidence intervals, and multiplicity-adjusted results for the 15 exploratory categorical association tests.

Marker	Variable	Test	Raw P	BH q	Effect size (95% CI)
PTEN	Age	Pearson chi-square	0.810	0.810	V=0.05 (0.00–0.18)
PTEN	Menopausal status	Fisher's exact	0.072	0.389	V=0.16 (0.00–0.32)
PTEN	T stage	Monte Carlo exact	0.596	0.810	V=0.09 (0.00–0.23)
PTEN	Nodal status	Fisher's exact	0.726	0.810	V=0.04 (0.00–0.20)
PTEN	Histological subtype	Monte Carlo exact	0.738	0.810	V=0.15 (0.00–0.26)
PTEN	Histological grade	Fisher's exact	0.423	0.810	V=0.07 (0.00–0.24)
PTEN	Fudan subtype	Monte Carlo exact	0.781	0.810	V=0.12 (0.00–0.22)
TMPRSS4	Age	Pearson chi-square	0.726	0.810	V=0.07 (0.00–0.20)
TMPRSS4	Menopausal status	Fisher's exact	0.278	0.704	V=0.10 (0.00–0.26)
TMPRSS4	T stage	Monte Carlo exact	0.282	0.704	V=0.13 (0.00–0.28)
TMPRSS4	Nodal status	Fisher's exact	0.078	0.389	V=0.15 (0.00–0.32)
TMPRSS4	Histological subtype	Monte Carlo exact	0.714	0.810	V=0.15 (0.00–0.26)
TMPRSS4	Histological grade	Fisher's exact	0.223	0.704	V=0.11 (0.00–0.28)
TMPRSS4	Fudan subtype	Monte Carlo exact	0.025	0.379	V=0.26 (0.00–0.39)
PTEN/TMPRSS4	Cross-marker association	Fisher's exact	0.736	0.810	V=0.04 (0.00–0.20)

Table S3 note: Fisher's exact test was used for 2 × 2 tables; Pearson's chi-square test for non-sparse larger tables; and fixed-margin Monte Carlo exact testing with 100,000 replicates for sparse larger tables. Effect size is Cramér's V with a 95% CI.

Supplementary Table S4. Unadjusted survival analyses in the 59-patient cohort.

Endpoint	Marker	Comparison	Events	Log-rank P	HR (95% CI)
OS	PTEN	Retained vs loss	3 (3 retained; 0 loss)	0.030	Not estimable
PFS	PTEN	Retained vs loss	8 total	0.276	2.20 (0.51–9.48)
OS	TMPRSS4	Positive vs negative	3 total	0.954	1.07 (0.10–11.84)
PFS	TMPRSS4	Positive vs negative	8 (0 negative; 8 positive)	0.034	Not estimable

Table S4 note: Analyses used available follow-up in the 59 patients with complete, verifiable outcome data. Conventional Cox HRs were not estimable when one comparison group had no events. Complete treatment data were unavailable, and multivariable Cox analysis was not performed because only three deaths and eight PFS events were available; all survival results are exploratory and unadjusted. OS, overall survival; PFS, progression-free survival; HR, hazard ratio; CI, confidence interval.

Supplementary Table S5. Unadjusted survival analyses in the TCGA-BRCA and METABRIC cohorts.

Cohort	End-point	Marker	Comparison	Events	Log-rank P	HR (95% CI)
TCGA-BRCA	OS	PTEN	Retained vs loss	16 (7 loss, 9 retained)	0.464	1.427 (0.526, 3.871)
TCGA-BRCA	PFS	PTEN	Retained vs loss	12 (5 loss, 7 retained)	0.592	1.332 (0.423, 4.187)
TCGA-BRCA	OS	TMPRSS4	Positive vs negative	11 (4 negative, 7 positive)	0.051	3.421 (0.861, 13.591)
TCGA-BRCA	PFS	TMPRSS4	Positive vs negative	9 (4 negative, 5 positive)	0.544	1.467 (0.392, 5.490)
METABRIC	OS	PTEN	Retained vs loss	113 (56 loss, 57 retained)	0.472	0.873 (0.603, 1.264)
METABRIC	PFS	PTEN	Retained vs loss	84 (43 loss, 41 retained)	0.599	0.892 (0.581, 1.369)
METABRIC	OS	TMPRSS4	Positive vs negative	116 (59 negative, 57 positive)	0.457	0.871 (0.604, 1.256)
METABRIC	PFS	TMPRSS4	Positive vs negative	90 (46 negative, 44 positive)	0.620	0.901 (0.596, 1.363)

Table S5 note: PTEN loss/retained and TMPRSS4 negative/positive groups correspond to the lowest and highest transcript-expression tertiles, respectively. Analyses were unadjusted and exploratory. OS, overall survival; PFS, progression-free survival; HR, hazard ratio; CI, confidence interval.
